# Supplementary material for: Analysis of Phenolic Compounds in Commercial Cannabis sativa L. Inflorescences Using UHPLC-Q-Orbitrap HRMS
Source: Molecules. 2020 Jan 31;25(3):631. doi: 10.3390/molecules25030631 (PMC7037164; doi:10.3390/molecules25030631)
Supplement: Supplementary file 1 [file molecules-25-00631-s001.pdf]

Supplementary materials:

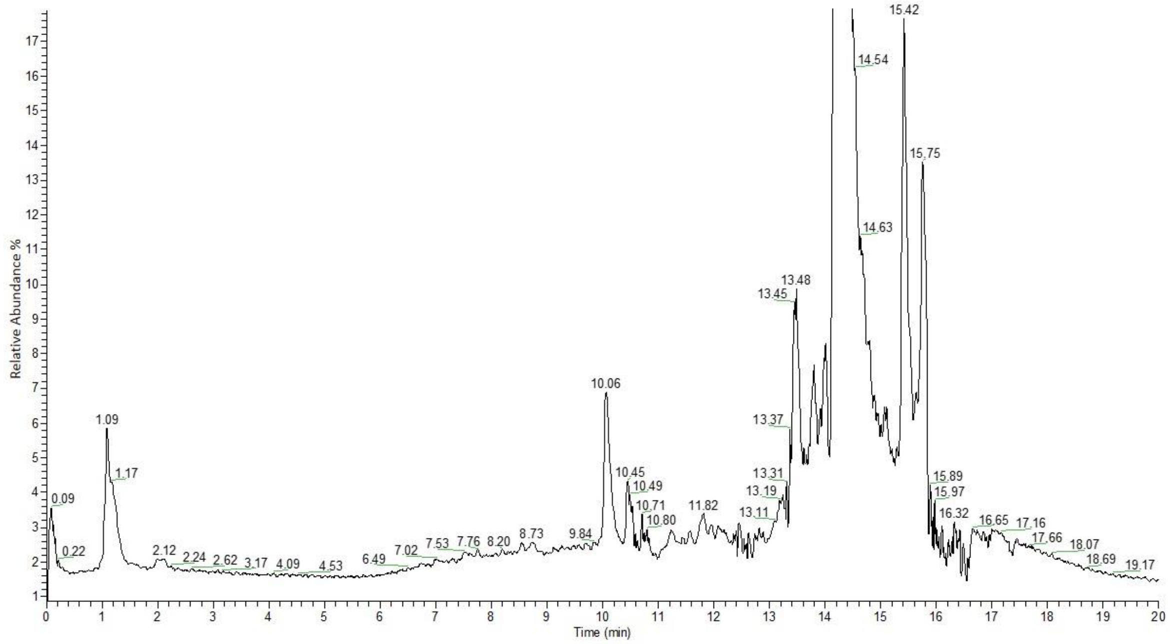

Supplementary Figure S1. Total Ion Chromatogram (TIC) of Carmagnola variety of C. *sativa* inflorescences through UHPLC-Q-Orbitrap HRMS.

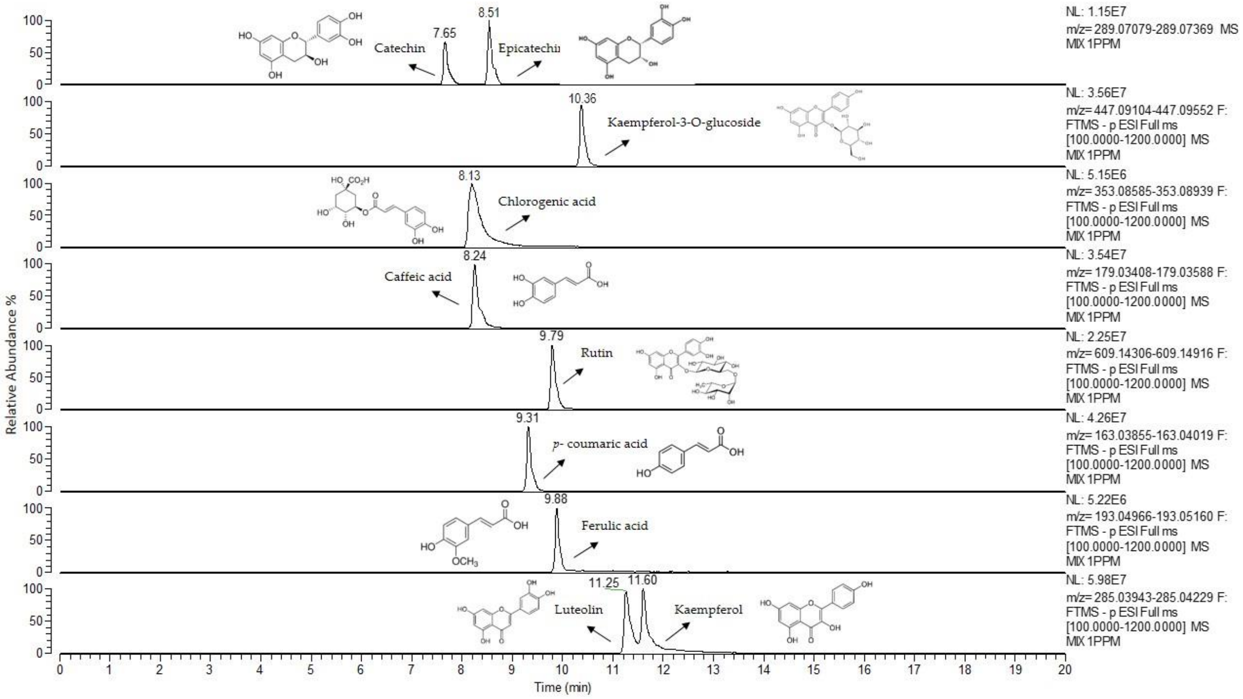

Catechin
7.65
8.51
Epicatechin
NL: 1.15E7 m/z= 289.07079-289.07369 MS MIX1PPM
10.36
Kaempferol-3-O-glucoside
NL: 3.56E7 m/z= 447.09104-447.09552 F: FTMS - p ESI Full ms [100.0000-1200.0000] MS MIX1PPM
8.13
Chlorogenic acid
NL: 5.15E6 m/z= 353.08585-353.08939 F: FTMS - p ESI Full ms [100.0000-1200.0000] MS MIX1PPM
8.24
Caffeic acid
NL: 3.54E7 m/z= 179.03408-179.03588 F: FTMS - p ESI Full ms [100.0000-1200.0000] MS MIX1PPM
9.79
Rutin
NL: 2.25E7 m/z= 609.14306-609.14916 F: FTMS - p ESI Full ms [100.0000-1200.0000] MS MIX1PPM
9.31
p- coumaric acid
NL: 4.26E7 m/z= 163.03855-163.04019 F: FTMS - p ESI Full ms [100.0000-1200.0000] MS MIX1PPM
9.88
Ferulic acid
NL: 5.22E6 m/z= 193.04966-193.05160 F: FTMS - p ESI Full ms [100.0000-1200.0000] MS MIX1PPM
11.25
11.60
Luteolin
Kaempferol
NL: 5.98E7 m/z= 285.03943-285.04229 F: FTMS - p ESI Full ms [100.0000-1200.0000] MS MIX1PPM
Relative Abundance %
Time (min)

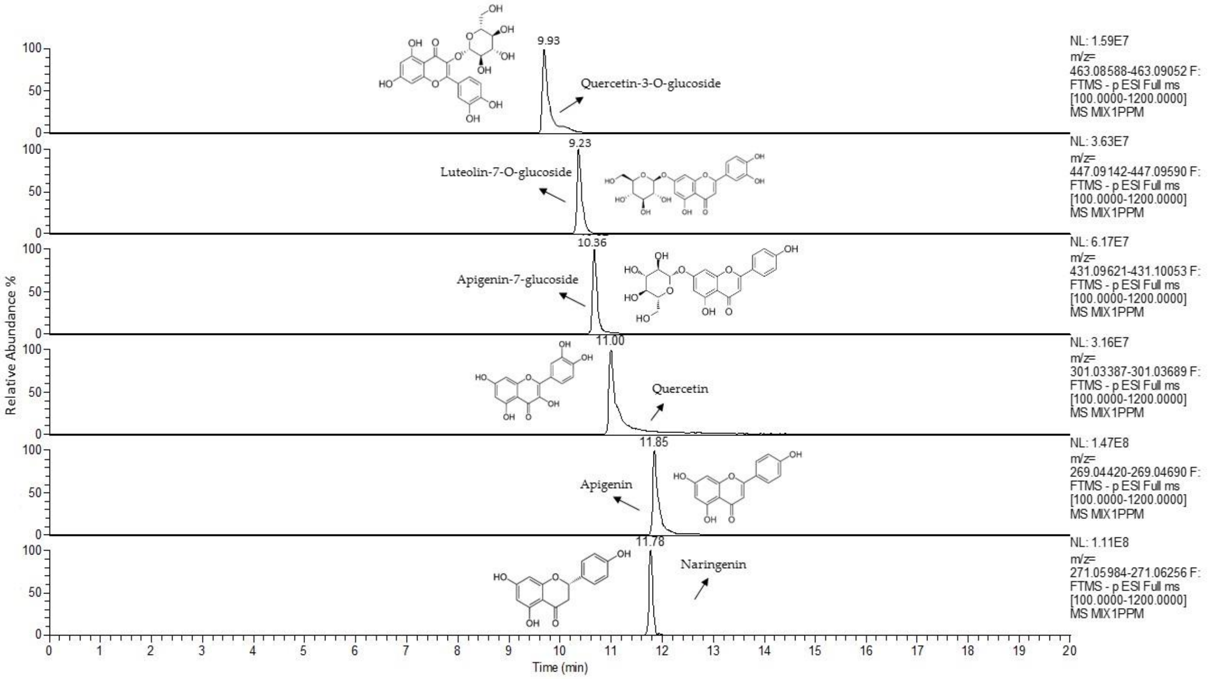

9.93
Quercetin-3-O-glucoside
NL: 1.59E7 m/z= 463.08588-463.09052 F: FTMS - p ESI Full ms [100.0000-1200.0000] MS MIX1PPM
9.23
Luteolin-7-O-glucoside
NL: 3.63E7 m/z= 447.09142-447.09590 F: FTMS - p ESI Full ms [100.0000-1200.0000] MS MIX1PPM
10.36
Apigenin-7-glucoside
NL: 6.17E7 m/z= 431.09621-431.10053 F: FTMS - p ESI Full ms [100.0000-1200.0000] MS MIX1PPM
11.00
Quercetin
NL: 3.16E7 m/z= 301.03387-301.03689 F: FTMS - p ESI Full ms [100.0000-1200.0000] MS MIX1PPM
11.85
Apigenin
NL: 1.47E8 m/z= 269.04420-269.04690 F: FTMS - p ESI Full ms [100.0000-1200.0000] MS MIX1PPM
11.78
Naringenin
NL: 1.11E8 m/z= 271.05984-271.06256 F: FTMS - p ESI Full ms [100.0000-1200.0000] MS MIX1PPM
Relative Abundance %
Time (min)

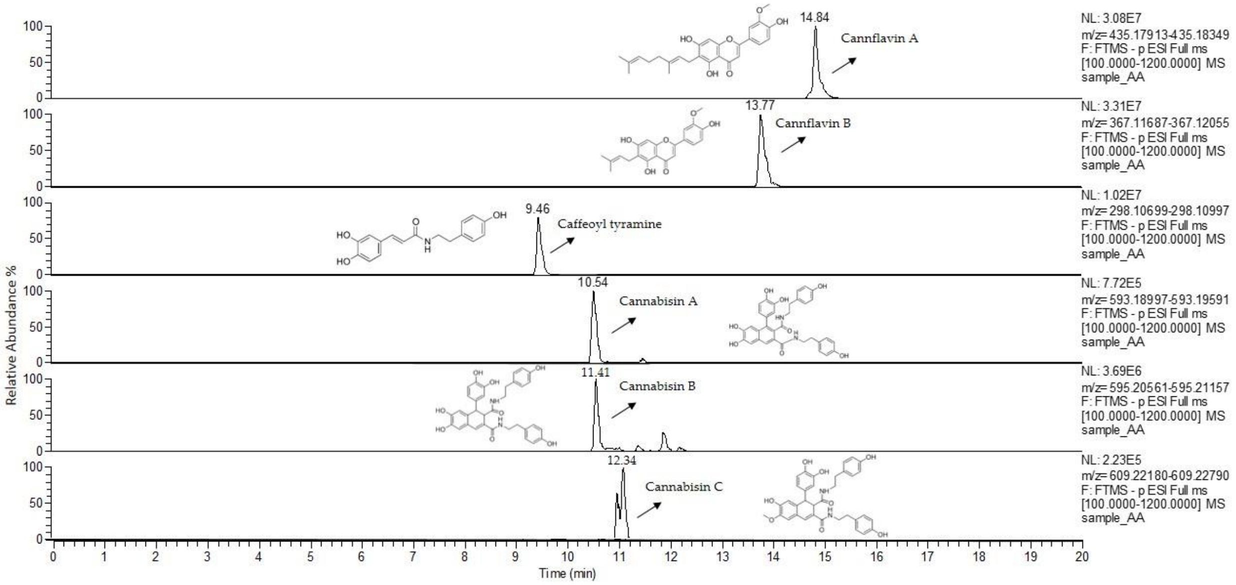

Supplementary Figure S2. Plots of twenty-two representative extracted ion chromatograms (EICs). The extracted EICs are represented with their specific *m*/*z*.

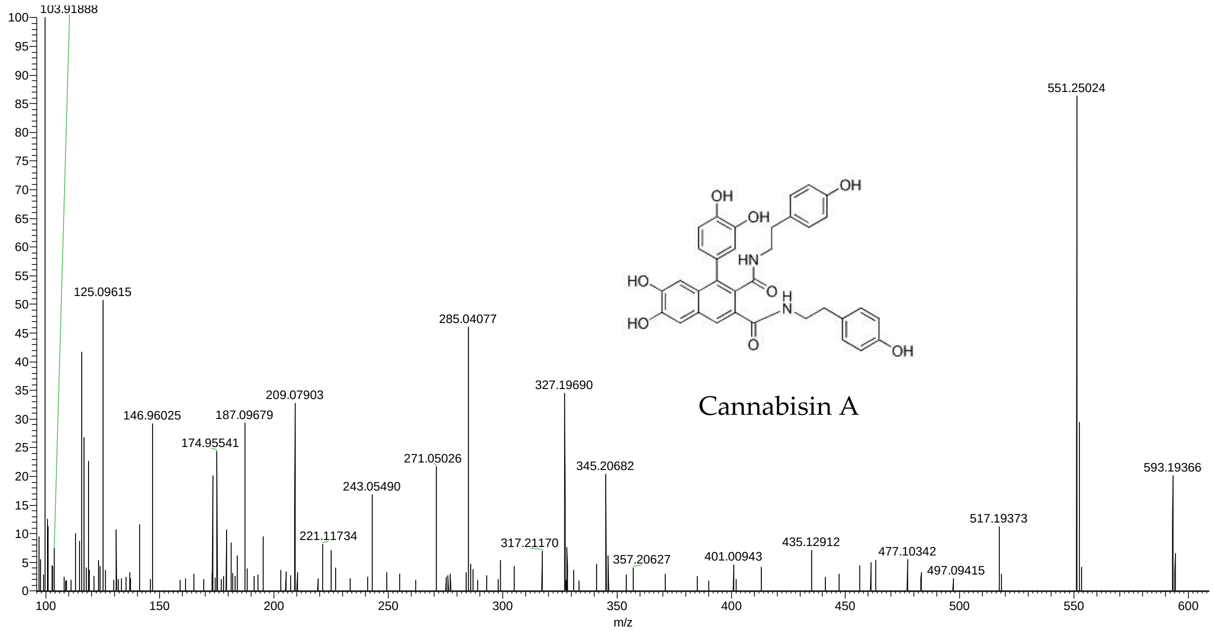
103.91888
125.09615
146.96025
174.95541
187.09679
209.07903
221.11734
243.05490
271.05026
285.04077
317.21170
327.19690
345.20682
357.20627
401.00943
435.12912
477.10342
497.09415
517.19373
551.25024
593.19366
m/z
OH
HO
HN
O
H
N
Cannabisin A

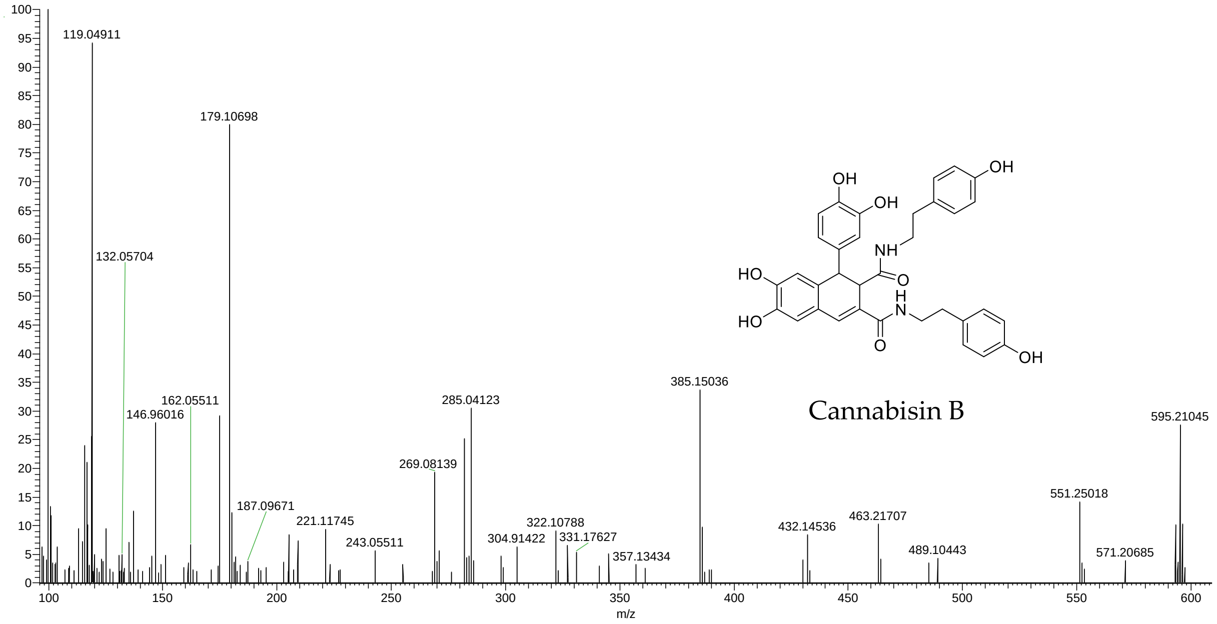
119.04911
132.05704
146.96016
162.05511
179.10698
187.09671
221.11745
243.05511
269.08139
285.04123
304.91422
322.10788
331.17627
357.13434
385.15036
432.14536
463.21707
489.10443
551.25018
571.20685
595.21045
m/z
OH
HO
NH
O
H
N
Cannabisin B

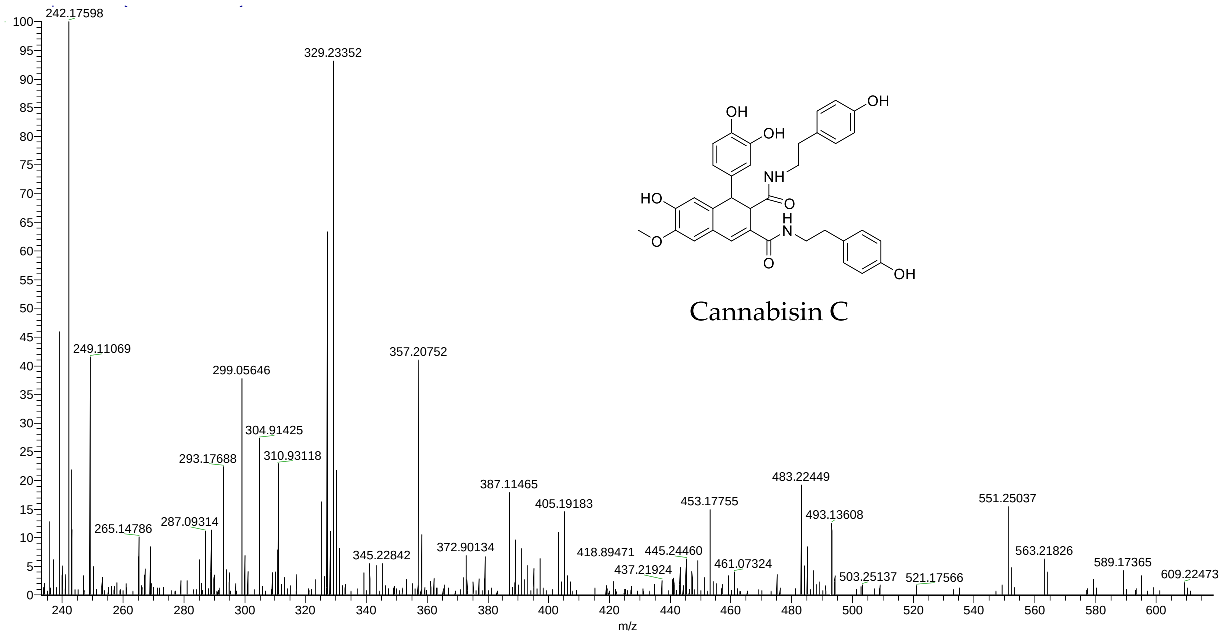

242.17598
329.23352
249.11069
299.05646
357.20752
304.91425
293.17688
310.93118
265.14786
287.09314
345.22842
372.90134
387.11465
405.19183
418.89471
437.21924
445.24460
453.17755
461.07324
483.22449
493.13608
503.25137
521.17566
551.25037
563.21826
589.17365
609.22473
m/z
Cannabisin C

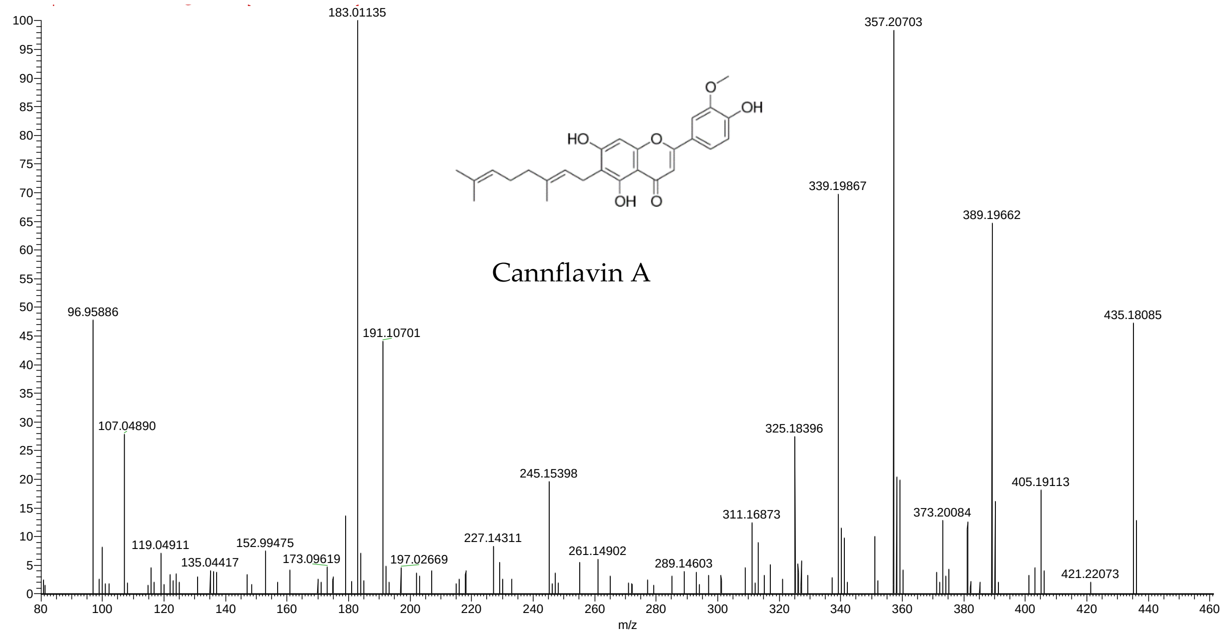

183.01135
357.20703
339.19867
389.19662
96.95886
191.10701
435.18085
107.04890
325.18396
245.15398
405.19113
373.20084
311.16873
119.04911
135.04417
152.99475
173.09619
197.02669
227.14311
261.14902
289.14603
421.22073
m/z
Cannflavin A

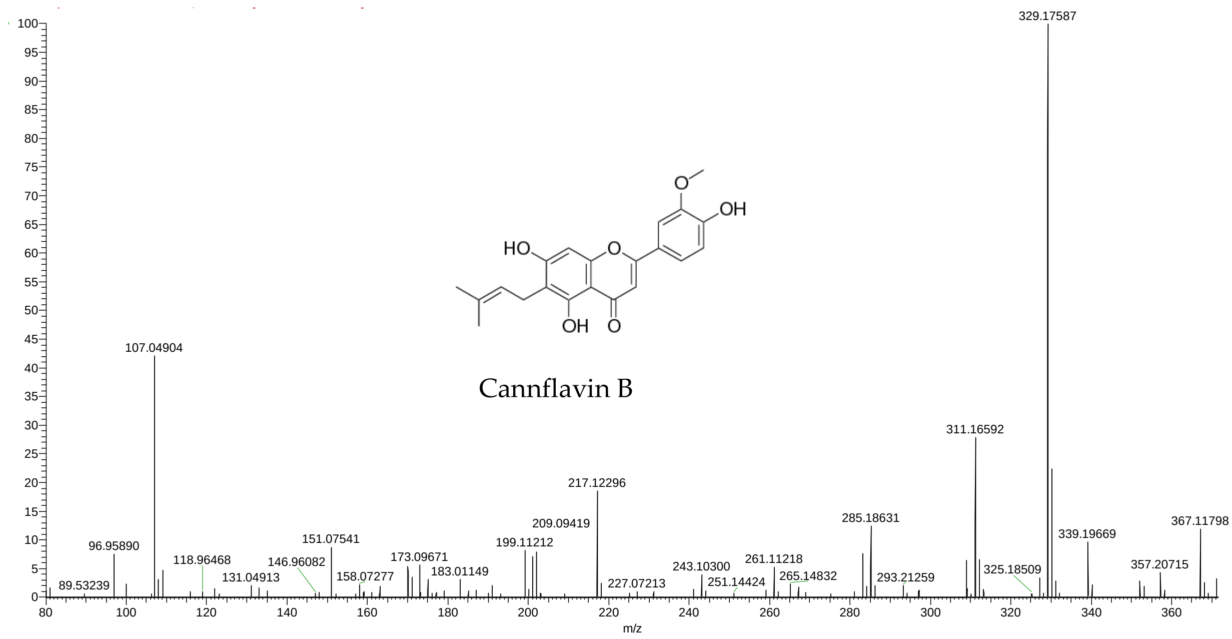
329.17587
107.04904
311.16592
217.12296
285.18631
367.11798
339.19669
209.09419
151.07541
96.95890
173.09671
199.11212
183.01149
118.96468
146.96082
243.10300
261.11218
131.04913
158.07277
265.14832
325.18509
357.20715
89.53239
227.07213
251.14424
293.21259
HO
OH
O
Cannflavin B
m/z

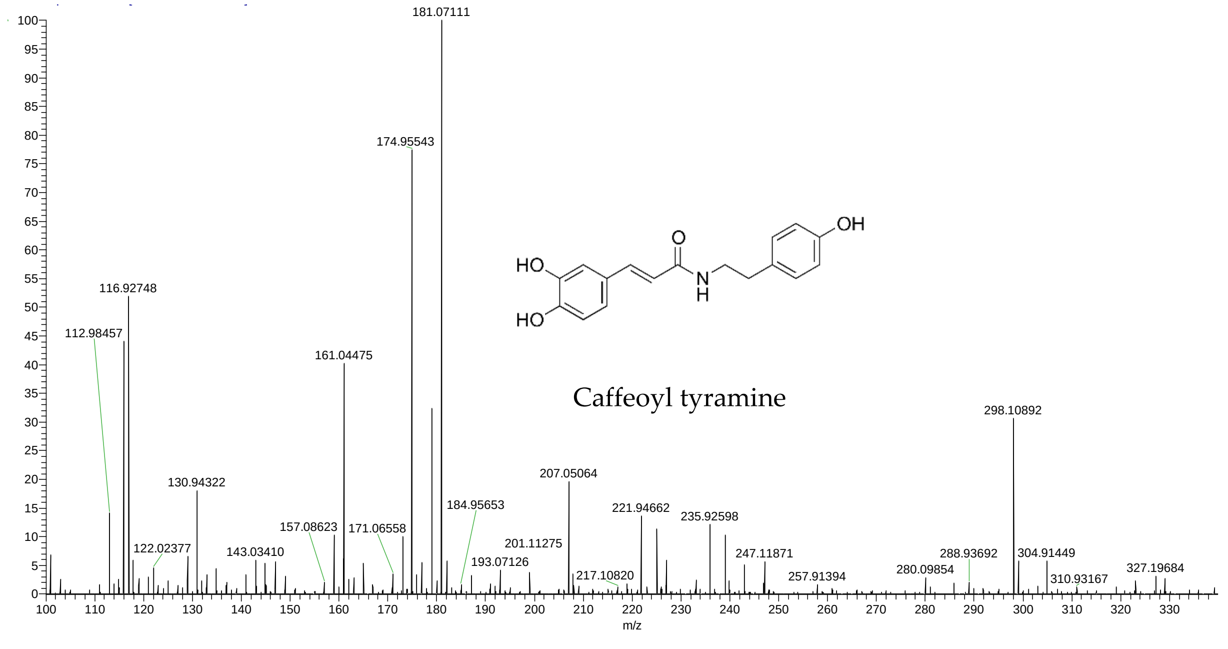
181.07111
174.95543
116.92748
112.98457
161.04475
298.10892
130.94322
207.05064
184.95653
221.94662
235.92598
157.08623
171.06558
122.02377
143.03410
201.11275
193.07126
247.11871
288.93692
304.91449
280.09854
327.19684
217.10820
257.91394
310.93167
HO
OH
O
N
H
Caffeoyl tyramine
m/z

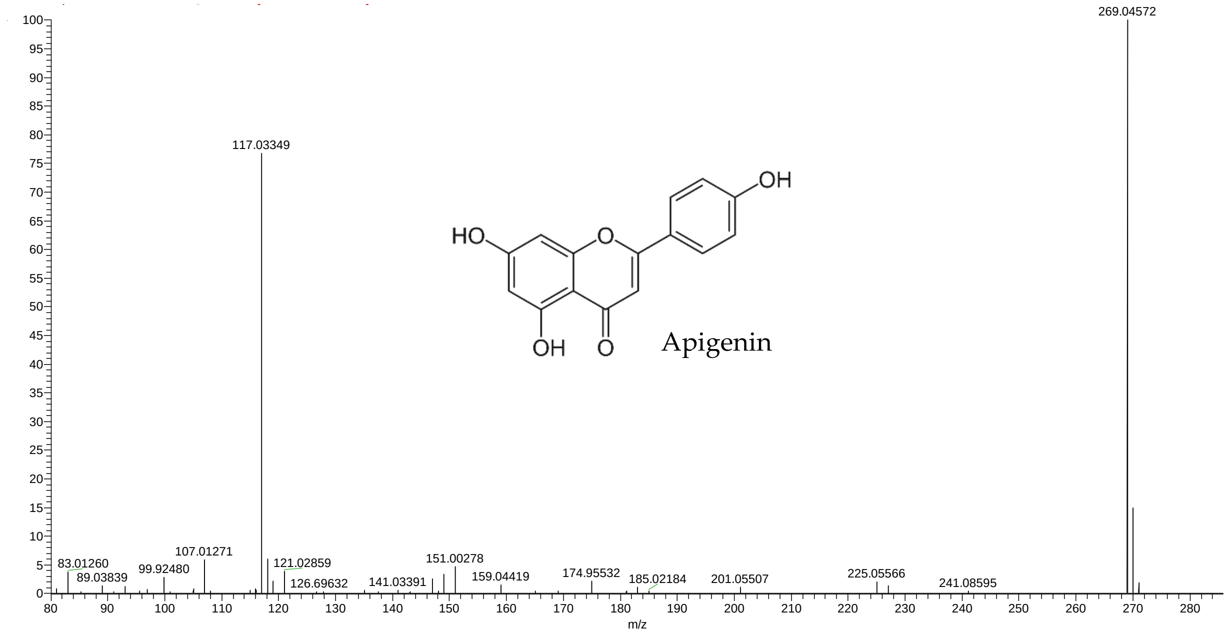

269.04572
117.03349
107.01271
83.01260
89.03839
99.92480
121.02859
126.69632
141.03391
151.00278
159.04419
174.95532
185.02184
201.05507
225.05566
241.08595
HO
OH
O
OH
O
Apigenin
m/z

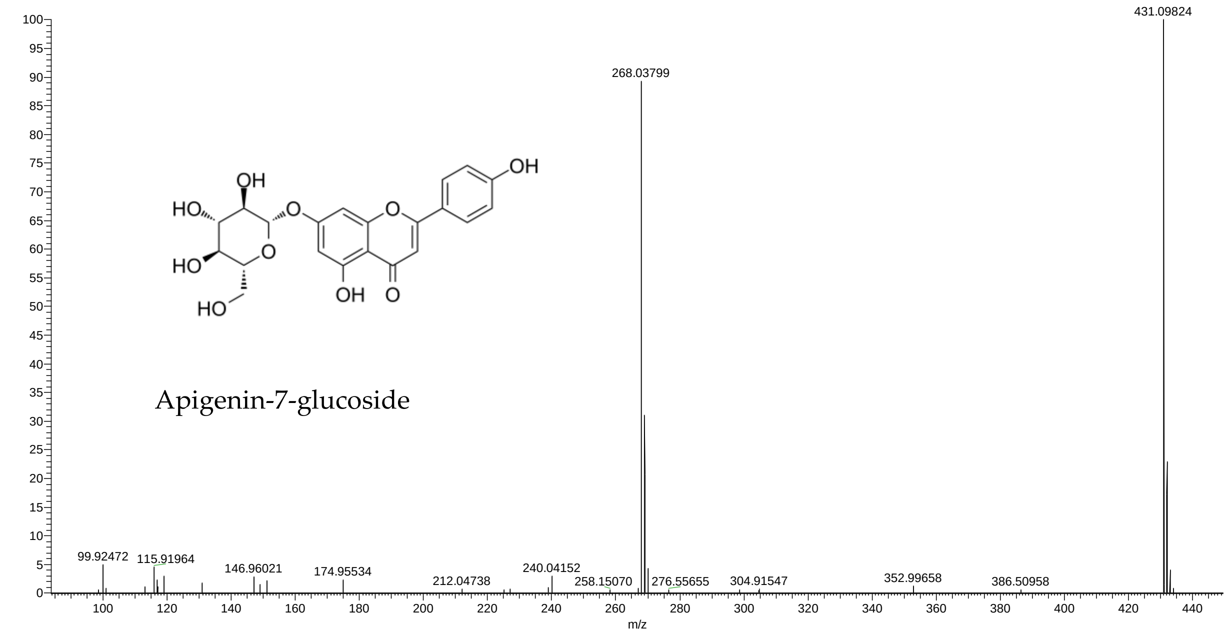

431.09824
268.03799
OH
HO
O
HO
O
HO
O
OH
O
OH
Apigenin-7-glucoside
99.92472
115.91964
146.96021
174.95534
212.04738
240.04152
258.15070
276.55655
304.91547
352.99658
386.50958
m/z

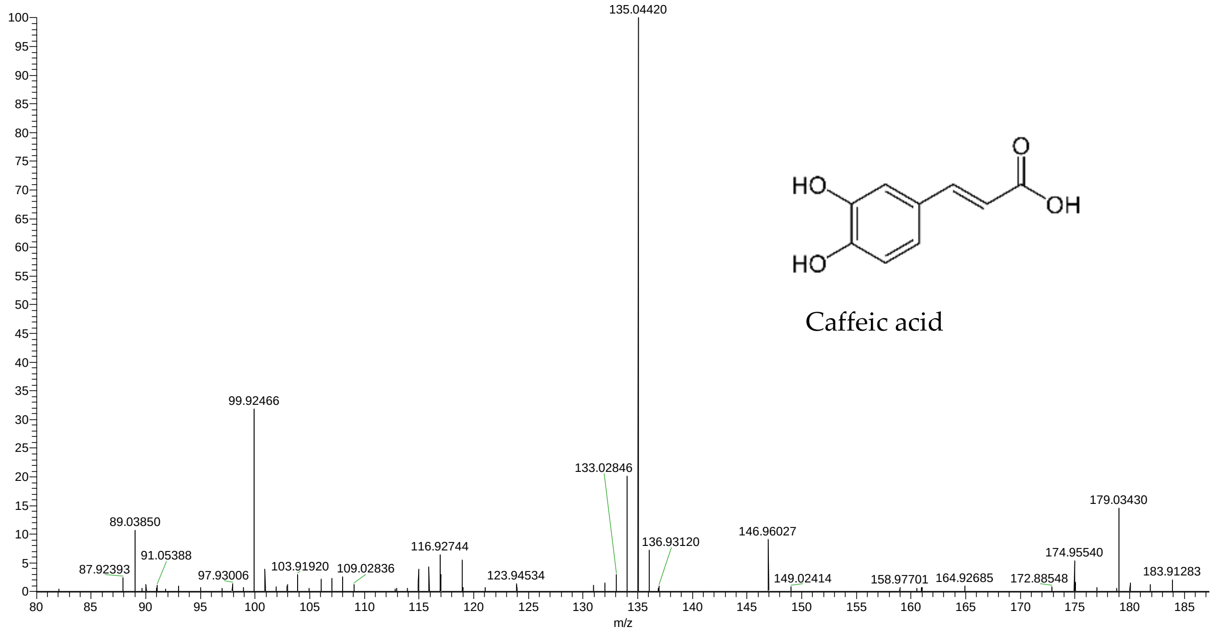
Caffeic acid
HO
HO
O
OH
135.04420
99.92466
133.02846
89.03850
87.92393
91.05388
97.93006
103.91920
109.02836
116.92744
123.94534
136.93120
146.96027
149.02414
158.97701
164.92685
172.88548
174.95540
179.03430
183.91283
m/z

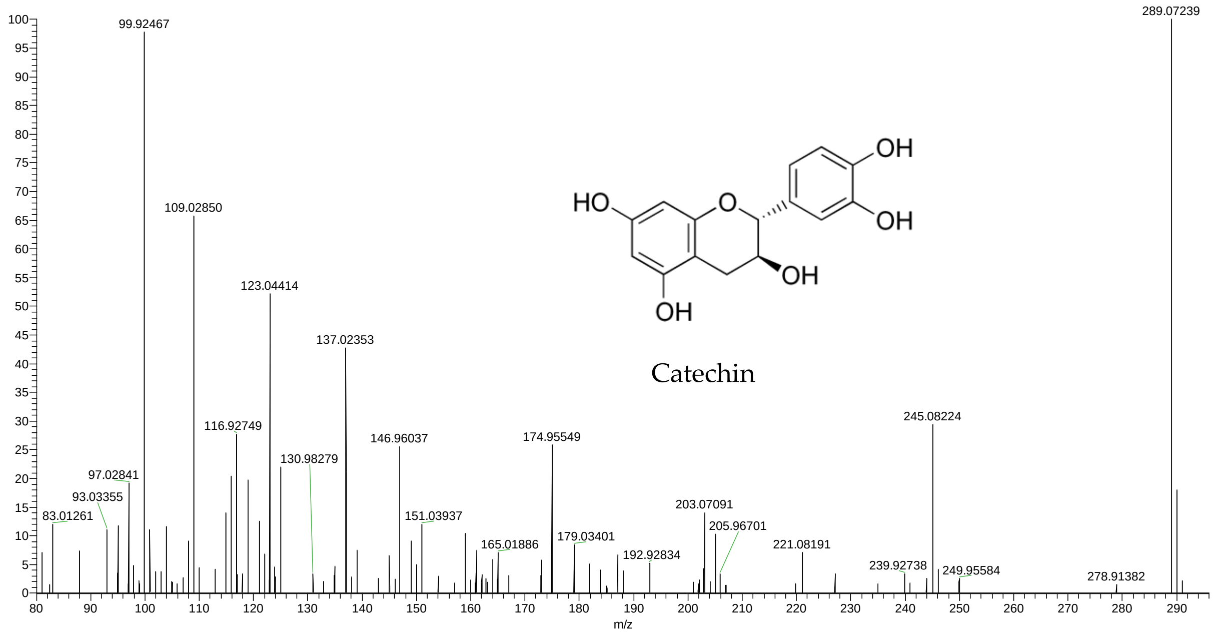
Catechin
HO
O
OH
OH
OH
OH
289.07239
99.92467
109.02850
123.04414
137.02353
116.92749
130.98279
146.96037
174.95549
245.08224
97.02841
93.03355
83.01261
151.03937
165.01886
179.03401
192.92834
203.07091
205.96701
221.08191
239.92738
249.95584
278.91382
m/z

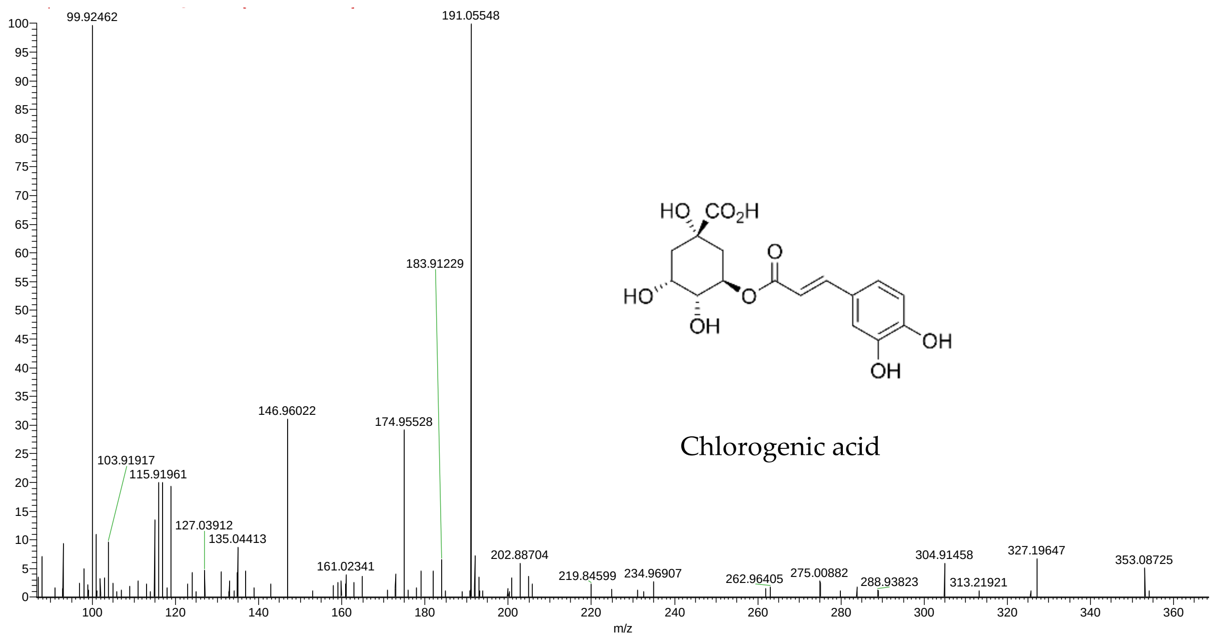
99.92462
191.05548
183.91229
146.96022
174.95528
103.91917
115.91961
127.03912
135.04413
161.02341
202.88704
219.84599
234.96907
262.96405
275.00882
288.93823
304.91458
313.21921
327.19647
353.08725
m/z
HO
CO_2H
O
HO
OH
OH
OH
Chlorogenic acid

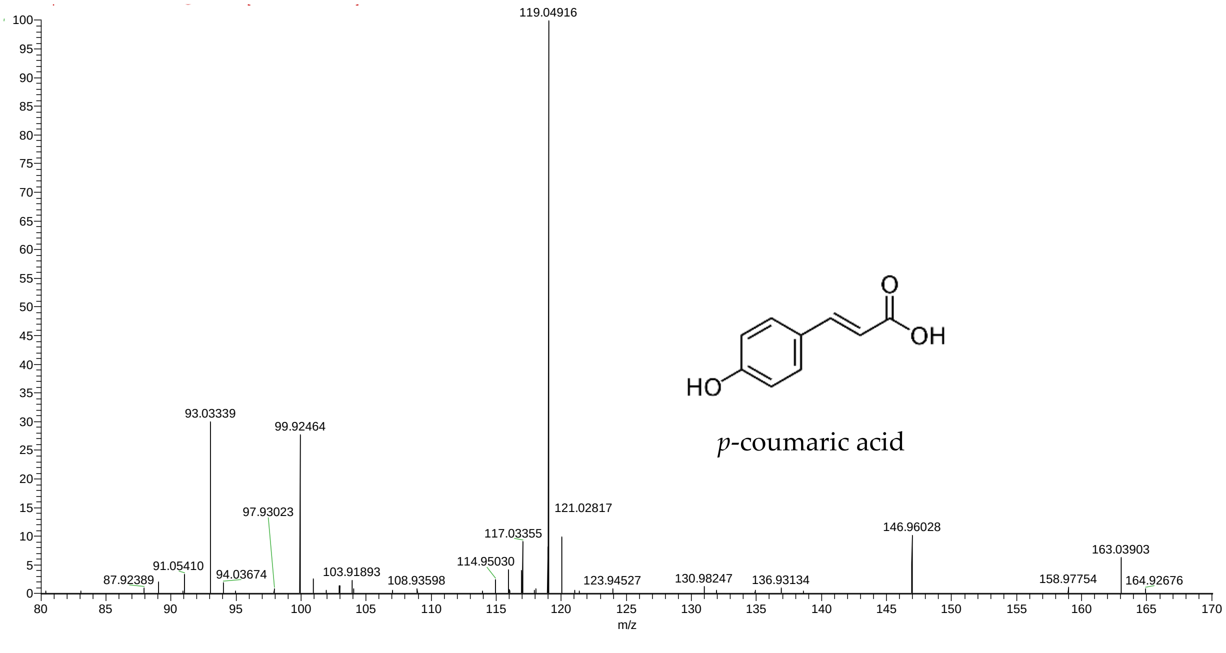
119.04916
93.03339
99.92464
97.93023
121.02817
117.03355
146.96028
114.95030
163.03903
91.05410
87.92389
94.03674
103.91893
108.93598
123.94527
130.98247
136.93134
158.97754
164.92676
m/z
O
OH
HO
p-coumaric acid

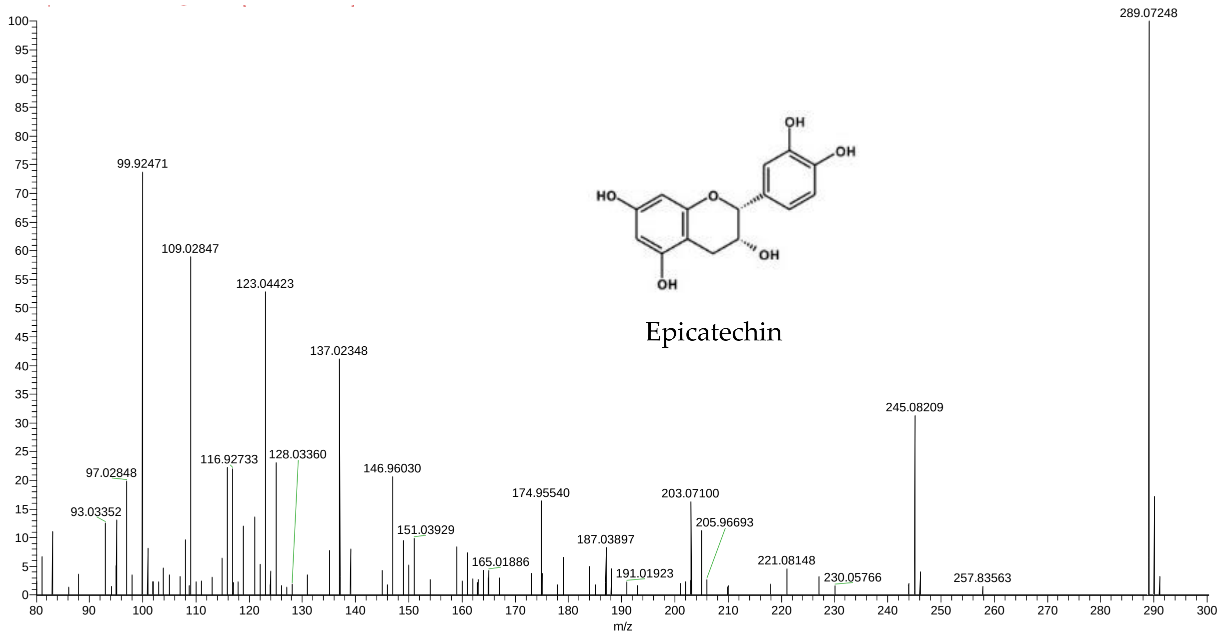
289.07248
99.92471
109.02847
123.04423
137.02348
245.08209
116.92733
128.03360
97.02848
146.96030
93.03352
174.95540
203.07100
187.03897
151.03929
205.96693
165.01886
221.08148
191.01923
230.05766
257.83563
m/z
Epicatechin

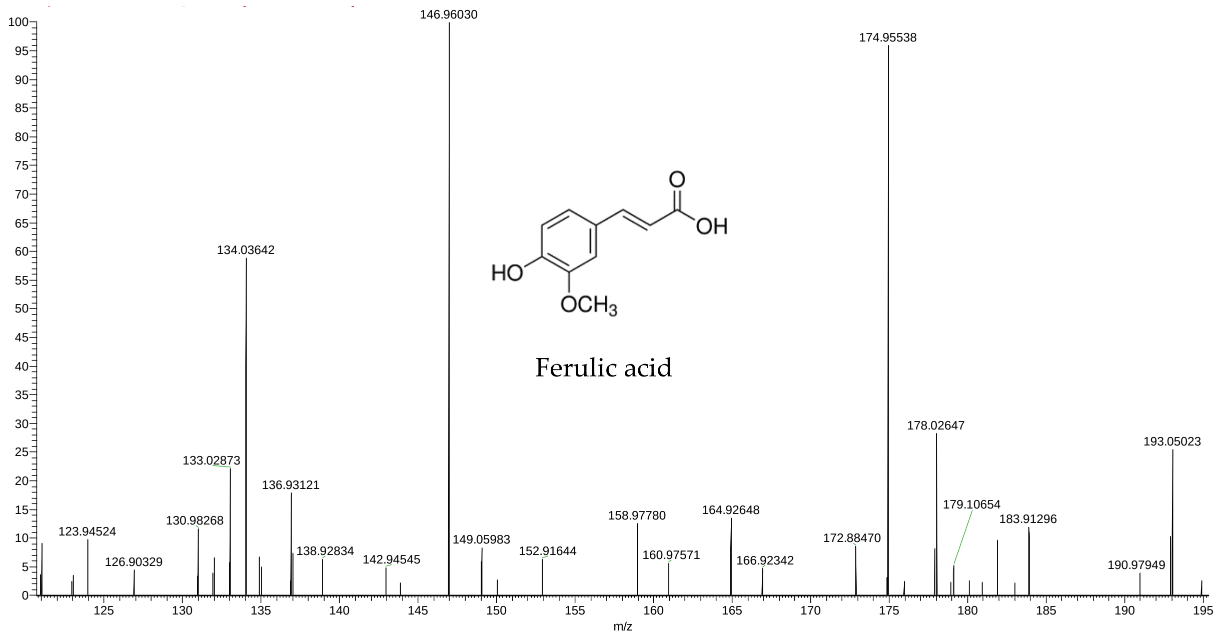
146.96030
174.95538
134.03642
178.02647
193.05023
133.02873
136.93121
179.10654
183.91296
158.97780
164.92648
123.94524
130.98268
149.05983
172.88470
152.91644
160.97571
138.92834
142.94545
126.90329
166.92342
190.97949
m/z
Ferulic acid

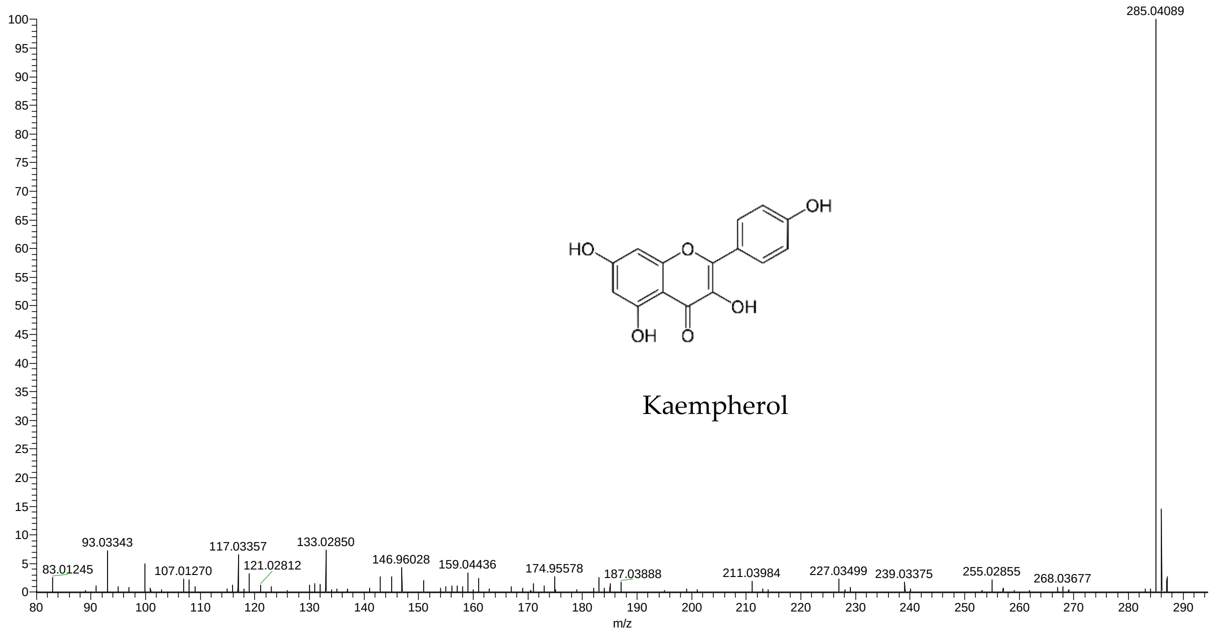
285.04089
93.03343
117.03357
133.02850
83.01245
107.01270
121.02812
146.96028
159.04436
174.95578
187.03888
211.03984
227.03499
239.03375
255.02855
268.03677
m/z
Kaempherol

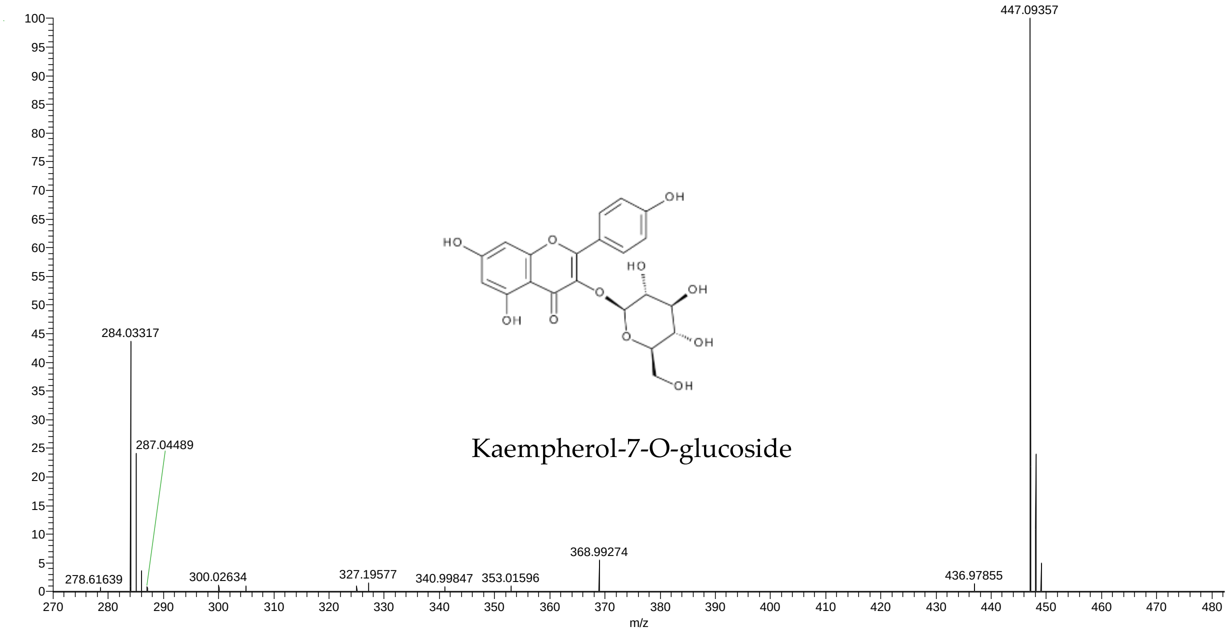
447.09357
284.03317
287.04489
278.61639
300.02634
327.19577
340.99847
353.01596
368.99274
436.97855
m/z
Kaempherol-7-O-glucoside

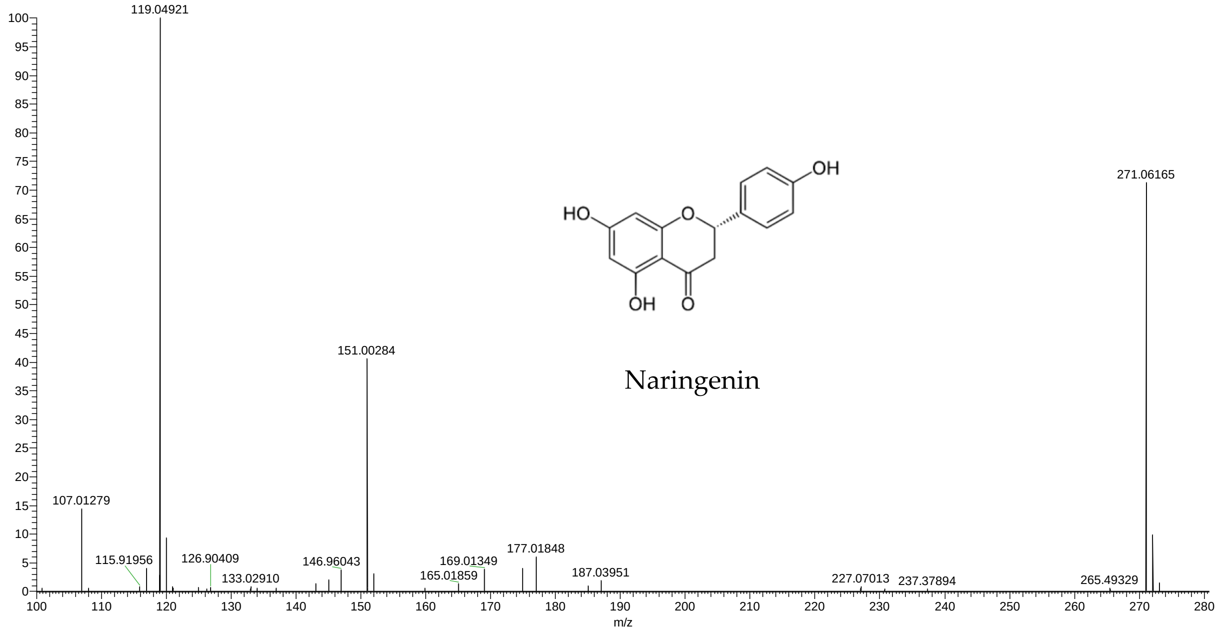
119.04921
271.06165
151.00284
107.01279
115.91956
126.90409
133.02910
146.96043
165.01859
169.01349
177.01848
187.03951
227.07013
237.37894
265.49329
HO
OH
OH
O
O
Naringenin
m/z

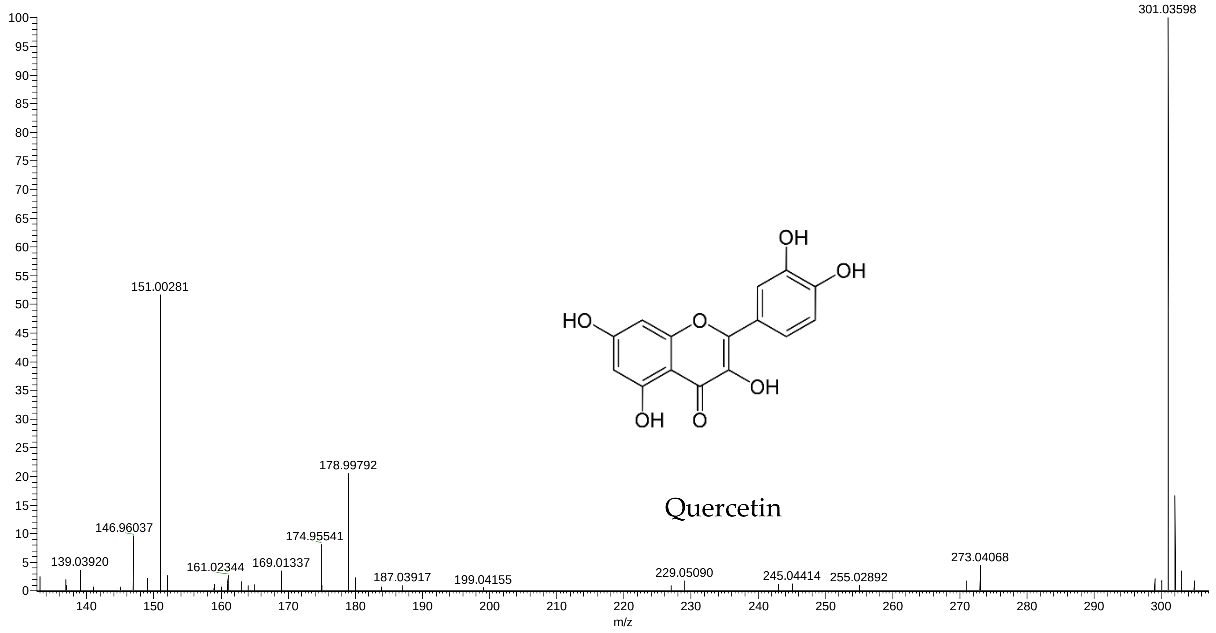
301.03598
151.00281
178.99792
146.96037
174.95541
139.03920
161.02344
169.01337
187.03917
199.04155
229.05090
245.04414
255.02892
273.04068
OH
OH
HO
O
OH
OH
O
Quercetin
m/z

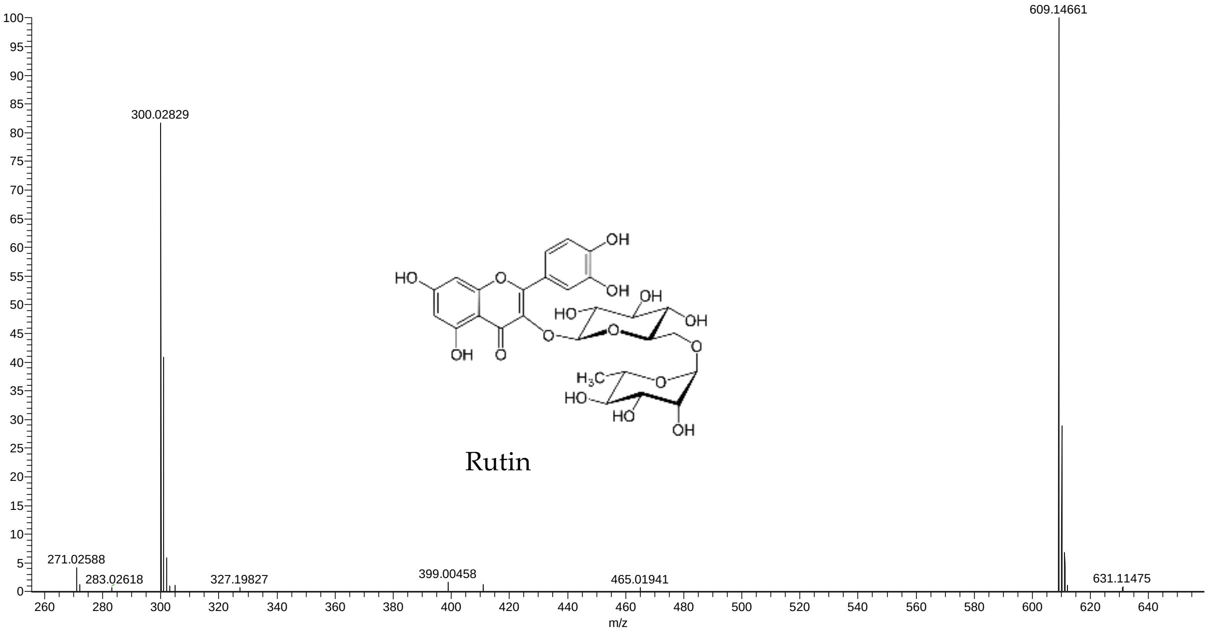
609.14661
300.02829
271.02588
283.02618
327.19827
399.00458
465.01941
631.11475
m/z
HO
OH
OH
OH
HO
OH
O
O
H_3C
HO
HO
OH
Rutin

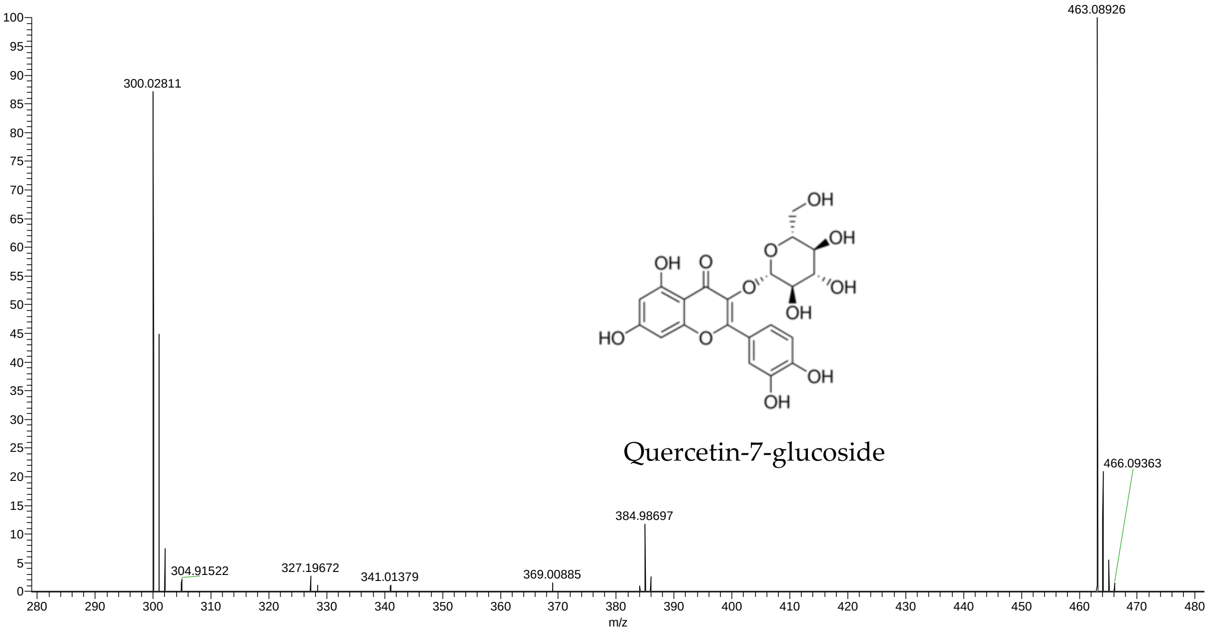
463.08926
300.02811
466.09363
384.98697
304.91522
327.19672
341.01379
369.00885
m/z
OH
O
OH
OH
OH
OH
HO
OH
OH
Quercetin-7-glucoside

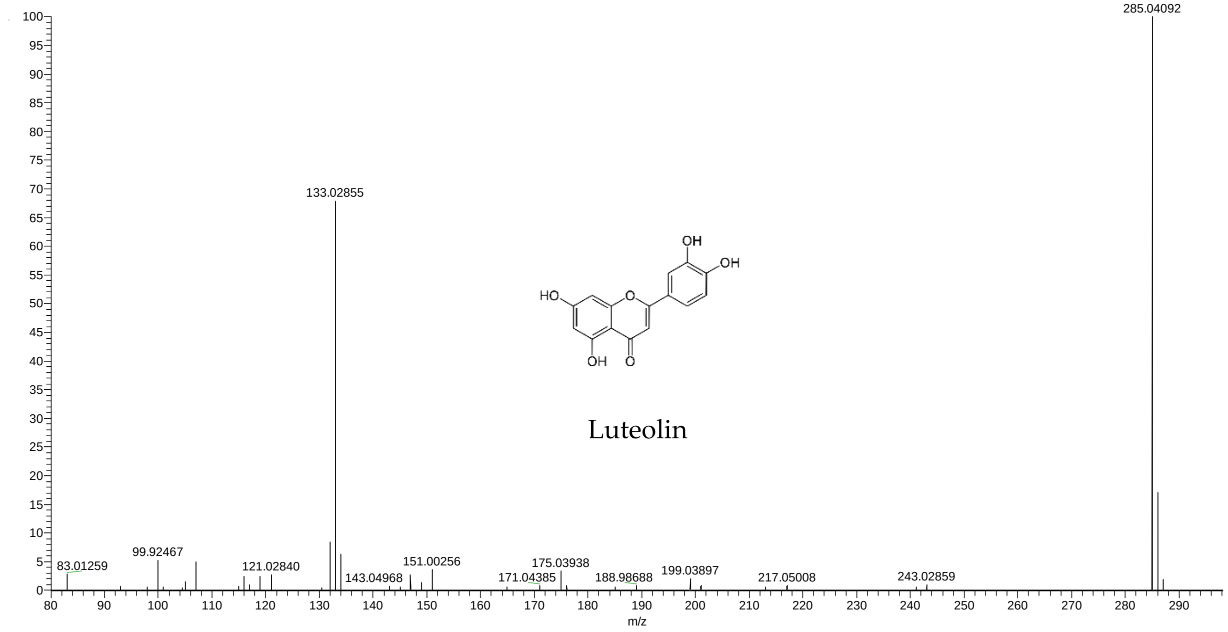

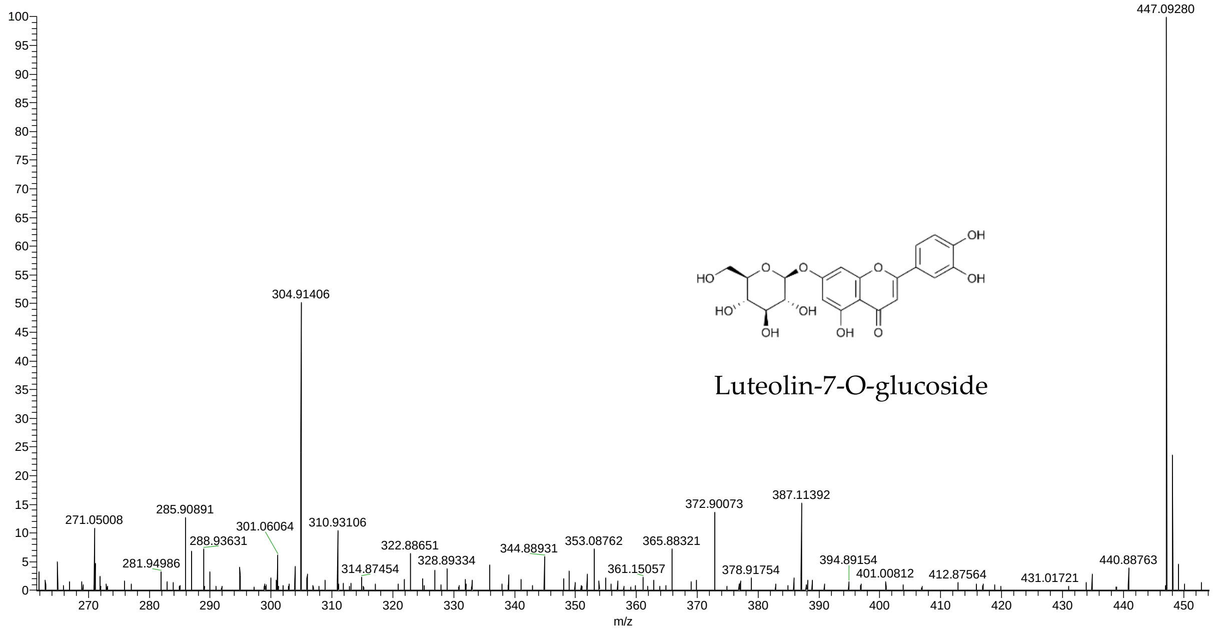

Supplementary Figure S3. MS/MS mass spectra of investigated compounds (*n=22*) extracted of C. *sativa* inflorescences.
